# Microplastics and Nanoplastics in Health Concerning Cellular Toxicity Mechanisms, Exposure Pathways, and Global Mitigation Strategies

**DOI:** 10.3390/life15091449

**Published:** 2025-09-16

**Authors:** Ruei-Hong Lin, Hao-Ting Chen, I-Ta Lee, Thi-Thuy-Tien Vo, Yung-Li Wang

**Affiliations:** 1 National Keelung Senior High School, Keelung City 205001, Taiwan; cht0905775708@icloud.com; 2 School of Dentistry, College of Oral Medicine, Taipei Medical University, Taipei City 11031, Taiwan; b202108058@gmail.com (H.-T.C.); itlee0128@tmu.edu.tw (I.-T.L.); 3 Faculty of Dentistry, Nguyen Tat Thanh University, Ho Chi Minh City 700000, Vietnam; vtttien@ntt.edu.vn

**Keywords:** microplastics, nanoplastics, oxidative stress, inflammation, genotoxicity, environmental policy, public health

## Abstract

Microplastics (MPs) and nanoplastics (NPs) have emerged as ubiquitous environmental contaminants that pose significant threats to human health, with multiple exposure pathways (e.g., ingestion and inhalation) contributing to systemic exposure. Although growing evidence highlights their biological effects, the underlying mechanisms by which these particles induce cellular dysfunction remain incompletely understood. This review synthesizes current knowledge on the MPs/NPs-induced cellular toxicity mechanisms, including investigations into cellular uptake pathways, disruption of molecular signaling, oxidative stress, inflammatory responses, and genotoxic effects. MPs/NPs contamination can arise from consumer products and clinical procedures, with estimated Daily Microplastic Emission (DME)-based national totals (India) ranging from ~0.36 to 74 billion particles/day across oral-care product categories. At the cellular level, MPs and NPs trigger interconnected toxicological cascades, including impaired endocytosis, mitochondrial dysfunction, chronic inflammation, genotoxicity, endoplasmic reticulum (ER) stress, and accelerated cellular senescence. These mechanisms act in concert to compromise epithelial barrier integrity. Overall, MPs/NPs present substantial risks to health through multiple interconnected pathways. Local and systemic effects are plausible across exposed tissues which may also serve as a gateway for systemic distribution by these contaminants. These findings highlight the urgent need for coordinated global efforts, including restrictions on intentionally added MPs, improvements in product design, development of advanced removal technologies, and implementation of clinical prevention strategies.

## 1. Introduction

The emergence of microplastics (MPs; particles < 5 mm) and nanoplastics (NPs; particles < 1 or 0.1 μm) as pervasive environmental pollutants represents one of the most pressing challenges in contemporary environmental health science. These synthetic polymer particles have infiltrated virtually all ecosystems examined to date, creating an unprecedented situation where human exposure has become virtually unavoidable [1,2].

Recent analytical advances have reported the presence of MPs/NPs contamination across multiple categories of oral healthcare products, dental materials, and environmental media that come into contact with oral tissues [3,4]. The extensive integration of plastic materials into modern dentistry including oral-care products, dental restorative and prosthodontic materials, orthodontic appliances, and clinical equipment has created multiple pathways for both intentional and unintentional MPs/NPs release directly into the oral environment [5]. The oral cavity represents a particularly critical interface in this exposure paradigm, functioning simultaneously as a primary entry portal for systemic distribution and as a site of direct toxicological impact where local cellular damage can have far-reaching physiological consequences [5]. From a clinical perspective, patients’ exposure is typically intermittent and product-associated (e.g., toothpaste, mouthwash, dental floss), whereas dental healthcare professionals experience cumulative and occupational exposure due to repeated handling of resin-based composites, polishing procedures, and aerosol-generating instrumentation. This occupational dimension highlights the importance of effective source control, high-volume evacuation, and appropriate personal protective equipment in dental settings. Simultaneously, environmental contamination of food, water, and even atmospheric particulates ensures continuous background exposure to MPs/NPs from external sources, creating a complex multi-source exposure scenario involving MPs and NPs that challenge traditional risk assessment approaches [1].

The biological significance of this exposure has become increasingly apparent as mechanistic studies reveal that MPs/NPs can induce profound cellular dysfunction through multiple interconnected pathways. Unlike traditional environmental toxicants that may target specific cellular processes, MPs and NPs appear to disrupt fundamental cellular machinery including membrane integrity, mitochondrial function, protein homeostasis, and genomic stability [6,7]. These effects are particularly concerning given the persistent nature of plastic particles, which cannot be metabolized or eliminated through normal biological processes, leading to potential bioaccumulation and chronic exposure scenarios; current human biomonitoring primarily detects MPs, with NPs likely under-detected due to analytical limits [1,6,7].

## 2. Sources and Pathways of Exposure to MPs/NPs

### 2.1. Consumer Product-Related Sources: Oral Healthcare Products and Dental Materials

The ubiquity of MPs/NPs contamination in oral healthcare products represents a paradigm shift in our understanding of intraoral, iatrogenic exposure risks in routine healthcare practices. Recent analytical studies using advanced spectroscopic techniques have reported MPs/NPs presence across multiple major categories of oral-care products, indicating that exposure can occur in the mouth via products intended to promote oral health [3,4,8]. Recent investigations employing Fourier-transform infrared (FTIR) spectroscopy and scanning electron microscopy have characterized product-level MPs/NPs contamination, with particle counts varying dramatically across product categories [4]. Toothpaste released polyethylene (PE) (~22%) and ethylene–vinyl acetate (EVA) (~78%) MPs, while toothbrushes shed polypropylene (PP) (~22%), with ~63% of particles < 100 µm; estimated intraoral annual exposure was ~1.183 × 10^6^ particles from toothpaste and ≥2.33 × 10^6^ from toothbrushes [3]. Evidence from commercial toothpaste shows extractable MPs identified via FTIR/microscopy and evaluated for environmental risk [8]. Direct product-level NP measurements within oral-care items remain scarce; however, oral ingestion of NPs via beverages has been quantified by hyperspectral stimulated Raman scattering microscopy—about 2.4 ± 1.3 × 10^5^ particles per liter of bottled water, ~90% of which are NPs [9]. In addition to healthcare products, non-medical items used intraorally can be sources: a 2025 pilot conference reported that chewing gum can release thousands of MPs particles into saliva during typical chewing (preliminary, conference abstract; not yet peer-reviewed) [10]. As shown in Table 1, the emphasis on oral cavity-relevant exposure sources reflect the dental and oral health perspective of this work, aiming to illustrate clinically significant pathways.

A particularly concerning discovery involves dental floss as a significant source of both MPs and chemical exposure in oral healthcare routines. Most conventional dental floss is manufactured from synthetic materials, primarily nylon or polyester, which can release microplastic particles during use. More alarmingly, many popular floss brands utilize polytetrafluoroethylene (PTFE), a type of Teflon, as a coating or material to facilitate smooth gliding between teeth [11]. PTFE belongs to the polyfluoroalkyl substances (PFASs) family, often termed forever chemicals due to their environmental persistence and bioaccumulation potential. Studies have demonstrated PFAS-related exposure can vary by brand/material; prior work reported higher serum PFAS in users of some PTFE flosses, while a 2025 cross-sectional analysis of U.S. adults found lower overall serum PFAS among floss users but a small increase in perfluorooctanoic acid (PFOA)—causality cannot be inferred, and brand-specific effects remain uncertain [11]. During flossing, the mechanical friction and stress applied to the floss material can cause transfer of PTFE/PFAS to the oral environment; direct MPs shedding into gingival tissues is plausible but currently under-documented and warrants further study. Lifecycle assessment comparing nylon-based versus silk-based floss indicates substantial environmental burdens for conventional petroleum-based floss systems, while material substitution (e.g., silk) can reduce impacts in several categories [12].

Morphological analysis reveals that fragments commonly dominate over fibers in many matrices, but proportions vary widely by matrix and method; fixed global percentages are inappropriate [13]. Analysis of five Indian toothpaste brands reported MPs predominantly <50 μm (down to ~1.5 μm) with notable polymer variability across products; FTIR/Atomic Force Microscopy (AFM) confirmed MPs and polymer identities [14].

Risk assessment calculations based on contamination data provide alarming projections of population-level exposure from oral healthcare products alone. According to a recent review, Daily Microplastic Emission (DME) models from a comprehensive Indian study project that mouthwash contributes the highest daily emissions at 74 billion particles/day nationally, while mouth freshener sprays contribute 0.36 billion particles/day. Toothbrushes themselves have been documented to release 30–120 particles per brush during use, further supporting quantitative risk assessment models. Individual-level Annual Microplastic Exposure (AME) calculations reveal that toothbrush use alone contributes 48,910 particles per person per year, with additional contributions from other oral-care products [15].

### 2.2. Dental Procedures and Clinical Material Degradation

Dental procedures involving mechanical manipulation of synthetic materials represent a significant but underrecognized source of acute microplastic exposure for both patients and healthcare workers. During routine procedures, such as composite restoration placement, adjustment, and polishing, substantial quantities of MPs are generated through these processes [16,17]. In addition, orthodontic elastics (rubber bands) used clinically have been experimentally shown to release both MPs/NPs during use [17]. These particles become airborne through dental handpiece spray systems and can remain suspended in clinical air for extended periods, creating inhalation exposure risks [18,19].

Quantitative characterization of resin-based composite (RBC) microparticles demonstrates substantial environmental release during clinical procedures. Grinding and polishing of direct-placement RBCs produced particles with a median diameter of 6.39 μm after 360 s agitation (9.55 μm for control materials) and a maximum specific surface area of 1290 m^2^/kg; most particles were <10 μm, and FTIR signatures remained identifiable after 12 months of water aging. These MPs mainly consist of bisphenol A-glycidyl methacrylate (bis-GMA)-based resins, glass fillers, and coupling agents, raising concerns regarding inhalation exposure and long-term ecological impacts [20]. Evidence for NPs generation during polishing or aerosolization remains preliminary and requires confirmation with nano-resolved methods. From a clinical perspective, patients’ exposure is typically intermittent and product-associated (e.g., toothpaste, mouthwash, dental floss) [15], whereas dental healthcare professionals’ exposure can be cumulative due to repeated handling of resin-based composites, polishing, and aerosol-generating procedures; therefore, source control and PPE are essential in clinical settings [16,18,19,20]. Regarding indoor MPs exposure in dental units, one field study reported higher airborne/settled MPs burdens in teaching hospitals than in private clinics, seasonal variability (higher in winter), and polyethylene terephthalate (PET) as a dominant polymer; sex-stratified risk differences were modest. Because reported metrics vary by sampling matrix (air vs. settled dust vs. deposition) and unit, we avoid mixing units or deriving annualized counts not provided in the original study [19].

**Table 1:** Oral cavity-relevant sources of MPs/NPs and exposure features.

Exposure Source	Polymer Type(s)	Particle Size Range	Reported Release Quantity
Toothpaste	PE, PP	1–500 µm	Up to 5000 particles/g product [8,14]
Mouthwash	PE fragments	10–200 µm	~74 billion particles/day (population estimate) [15]
Toothbrush bristles	Nylon-6, PE	10–500 µm	30–120 particles per brushing [15]
Dental floss	Nylon, polytetrafluoroethylene (PTFE)	1–100 µm	Release during flossing detected [11]
Orthodontic rubber bands	Polystyrene (PS), Elastomers	100 nm–10 µm	Detectable MPs/NPs release [17]
Dental resin composites	Methacrylate-based polymers	0.5–20 µm	Resin microparticles during polishing [20]
Denture wear/procedures	Acrylic resins (PMMA)	1–50 µm	Release observed in clinical simulations [16]
Dental aerosols (procedures)	Mixed polymeric debris	<1 µm to >10 µm	High concentrations in aerosols [18,19]

### 2.3. Environmental Contamination Pathways

Environmental MPs and NPs enter the oral cavities through multiple dietary pathways that operate continuously throughout daily life, creating complex exposure scenarios that vary significantly based on geographical location, dietary patterns, and food preparation practices. Primary contamination occurs during food production, with agricultural soils increasingly contaminated by microplastics from sewage sludge application, atmospheric deposition, and plastic mulch degradation [21,22].

A recent survey of 130 seafood products from the German retail market quantified MPs with concentrations from 0 to 183 MP/g wet weight (median ~0.9 MP/g), with 97% <150 μm; canned fish had the highest levels (median ~2.4 MP/g). Only 16% of products exceeded pyrolysis–gas chromatography–mass spectrometry (pyrolysis-GC–MS) detection limits (polymers identified included PS, PET, PP). Estimated annual dietary intake via seafood was ~16,500 MPs per capita, underscoring packaging/processing as major contamination sources [23]. Similarly, analysis of 271 marine fish from 32 species in the Beibu Gulf, revealing a 93.7% detection rate of MPs with an average abundance of ~1.02 ± 0.18 items/individual; PET and PP dominated, and most particles were fibers/fragments < 1 mm [24].

Inhalation of airborne MPs represents a direct exposure pathway to oral and respiratory tissues via deposition on oral surfaces during breathing, speaking, and eating. Urban environments are particularly rich in MPs and NPs due to tire wear particles, textile fiber shedding, and general plastic degradation processes. Indoor air contamination can exceed outdoor levels, with synthetic textiles/furnishings as continuous fiber sources. Reported indoor fallout/deposition rates are on the order of 1586–11,130 particles/m^2^/day in some urban indoor environments; values vary widely by method and site [25].

## 3. Cellular and Molecular Mechanisms of Toxicity

### 3.1. Cellular Uptake Mechanisms and Barrier Dysfunction

**Figure 1:**
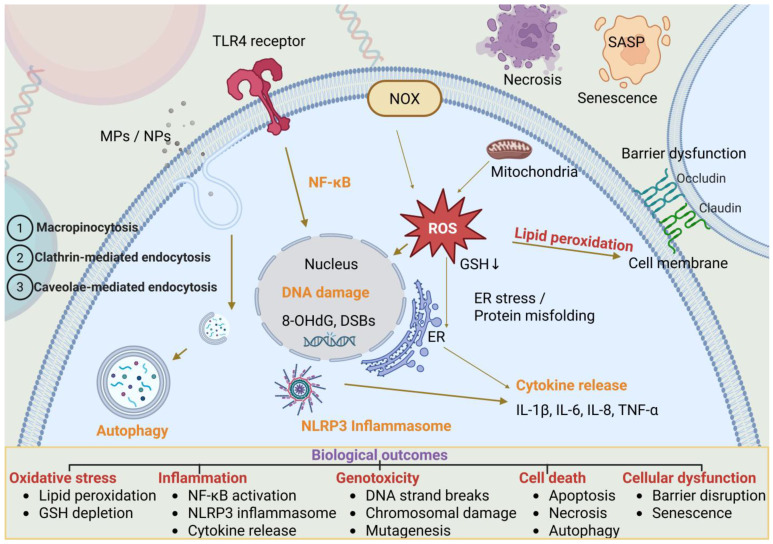
Cellular and molecular mechanisms of MPs/NPs-induced toxicity.

The cellular internalization of MPs/NPs involves sophisticated endocytic mechanisms (Figure 1) that demonstrate remarkable size selectivity and polymer specificity. Clathrin-mediated endocytosis represents the primary uptake pathway for particles in the 50–200 nm size range, with uptake efficiency generally decreasing as particle size increases [26,27,28,29,30,31]. This size-dependent uptake reflects the physical constraints of clathrin coated pit formation and the energetic requirements for membrane invagination. Larger particles (>500 nm) are mainly internalized via macropinocytosis and phagocytosis, processes that require significant cellular energy expenditure and may also interfere with membrane trafficking in certain contexts [27].

MPs/NPs contact the epithelium and enter via macropinocytosis, clathrin- and caveolae-mediated endocytosis. Recognition (e.g., Toll-like receptor (TLR)4) and NOX activation together with mitochondrial dysfunction elevate ROS, driving lipid peroxidation and GSH depletion. In parallel, ER stress/protein misfolding develops. ROS and lysosomal stress activate NF-κB and assemble the NLRP3 inflammasome, triggering cytokine release (IL-1β, IL-6, IL-8, TNF-α). Nuclear injury includes 8-OHdG(8-hydroxy-2’-deoxyguanosine) and γ-H2AX-marked double-strand breaks (DSBs). Disruption of tight-junction proteins (occludin, claudin) causes barrier dysfunction. These pathways lead to oxidative stress, inflammation, genotoxicity, cellular dysfunction (including senescence/senescence-associated secretory phenotype (SASP) and barrier disruption), and cell death (apoptosis, necrosis, autophagy).

Caveolin-mediated endocytosis is frequently implicated in the uptake of NPs in vitro [28,29], with particles < 50 nm showing preferential uptake through caveolar pathways that can in some cases delay early lysosomal routing; however, many studies still observe particles eventually localized to lysosomes. The caveolin-mediated pathway also provides access to transcytosis routes that might enable particle transport across epithelial barriers, though direct evidence for oral epithelial translocation to systemic circulation remains limited [28].

Surface functionalization modulates PS-NPs uptake and cytotoxicity in macrophages in vitro [29]. Carboxyl- and amino-modified PS-NPs were synthesized and tested in mouse macrophages. The results showed that the carboxylated NPs exhibited the highest cellular uptake and the most evident toxic effects, compared to the pristine or amino-modified forms. This enhanced uptake is likely due to stronger interactions with cell surface receptors, and environmental surface modifications substantially influence NPs behavior in cells [29]. NPs (e.g., PS-NPs) can be efficiently internalized through clathrin- and caveolae-mediated pathways and undergo transcytosis, highlighting their potential to cross epithelial barriers [26,27,28,29]. Epithelial barrier dysfunction has been observed after exposure to plastic particles in diverse epithelial models and in the mouse intestine [30,31]. Studies have demonstrated reduced expression of occludin and claudin-1 following MPs/NPs exposure, with consequent decreases in transepithelial electrical resistance (TEER) and increased paracellular flux of marker molecules [31]. Given their smaller size and higher uptake efficiency, NPs may more readily undergo transepithelial transport when tight-junction integrity is compromised [28,30,31].

### 3.2. Oxidative Stress and Mitochondrial Dysfunction

Oxidative stress represents a central mechanism of MPs/NPs toxicity that underlies many of the downstream cellular effects observed in exposed tissues. Multiple cellular sources contribute to enhanced ROS production following exposure to MPs/NPs, creating complex oxidative stress patterns that can overwhelm cellular antioxidant defenses [32,33,34,35,36,37,38]. The generation of ROS occurs through both direct particle–cell interactions and secondary effects mediated by stress signaling cascades [33].

Nicotinamide adenine dinucleotide phosphate (NADPH) oxidase (NOX) activation occurs through direct particle interaction with membrane-bound enzyme complexes and through inflammatory signaling cascades that upregulate oxidase expression and activity. This enzyme family represents a major source of superoxide radicals that can be rapidly converted to other reactive species including hydrogen peroxide and hydroxyl radicals. In lung and epithelial models, NPs induce ROS production and promote EMT and inflammatory signaling at lower thresholds compared with MPs [32,33]. In a uterine mouse model, PS-MPs activated the TLR4/NOX2 axis with downstream oxidative and fibrotic signaling [34].

Mitochondrial-derived ROS generation represents another critical source of oxidative stress, with evidence showing that MPs/NPs can impair mitochondrial membrane potential, disrupt electron transport, and promote ROS leakage; involvement of electron transport chain complexes such as I and III have been suggested but requires further confirmation [35]. This mitochondrial ROS generation is particularly concerning as it occurs in close proximity to mitochondrial DNA, which lacks the extensive repair mechanisms available for nuclear DNA. Studies using confocal microscopy with mitochondria-specific ROS indicators (e.g., MitoSOX) have reported that MPs/NPs exposure induces rapid increases in mitochondrial superoxide production, preceding detectable changes in cellular viability [36]. NPs can disrupt electron transport chain function and increase oxidative stress near mtDNA, enhancing risk of mitochondrial and nuclear damage [32,36].

Cellular antioxidant systems show complex responses to MPs/NPs exposure, with both adaptive upregulation and maladaptive suppression observed depending on exposure conditions and cell types. In particular, glutathione system dysfunction represents a critical component of MPs/NPs-induced oxidative stress, with particles causing depletion of reduced glutathione (GSH) and impairment of glutathione peroxidase and glutathione reductase activities [37,38]. The depletion of GSH reserves compromises the cell’s ability to neutralize hydrogen peroxide and lipid peroxides, creating conditions for oxidative damage to accumulate in cellular macromolecules. Such antioxidant imbalance has also been observed under NP exposure, indicating a stronger size-dependent amplification of oxidative damage [32,36].

### 3.3. Inflammatory Response and Immune System Dysregulation

The recognition of MPs/NPs as foreign materials by the innate immune system represents a critical early step in the inflammatory cascade that can have far-reaching consequences for tissue homeostasis and systemic health. Toll-like receptor (TLR) signaling pathways appear to play central roles in MPs/NPs recognition, with TLR4 showing frequent involvement across models [39,40,41,42,43,44]. The engagement of pattern recognition receptors by MPs/NPs initiates signaling cascades that culminate in transcriptional activation of inflammatory genes.

The activation of TLR signaling leads to nuclear factor-κB (NF-κB) translocation and subsequent upregulation of pro-inflammatory gene expression. This transcriptional response includes increased production of interleukin-1β (IL-1β), interleukin-6 (IL-6), tumor necrosis factor-α (TNF-α), and other inflammatory mediators that can recruit immune cells and amplify local inflammatory responses [41,42]. Such NF-κB-mediated responses have been observed in both mammalian and aquatic models, supporting the generality of this pathway.

The inflammasome pathway represents another critical component of the innate immune response to MPs/NPs. NLR family pyrin domain containing 3 (NLRP3) inflammasome activation has been documented following exposure to various plastic particles (MPs/NPs), leading to caspase-1 activation and subsequent IL-1β and IL-18 processing and secretion. The activation of inflammasomes by MPs/NPs appears to involve lysosomal destabilization following particle phagocytosis, with subsequent release of cathepsins into the cytoplasm triggering inflammasome assembly. This pathway is relevant for barrier epithelia with resident macrophages and dendritic cells [43].

Chronic exposure to MPs/NPs can lead to significant alterations in adaptive immune function that may have long-term consequences for immune surveillance and disease susceptibility. MPs/NPs can modulate dendritic-cell antigen presentation and costimulatory signals in vitro, with direction and magnitude depending on particle size and surface chemistry [44].

The persistent nature of plastic (MPs/NPs) particles in tissues creates conditions for chronic inflammatory responses that differ qualitatively from acute inflammatory reactions. Unlike biodegradable inflammatory stimuli that is eventually cleared, plastic particles can remain in tissues indefinitely, providing continuous inflammatory signaling. This chronic inflammation can lead to tissue remodeling, fibrosis, and creation of pro-carcinogenic microenvironments. Tissues examined after prolonged plastic particle exposure often show persistent inflammatory infiltrates and matrix remodeling [45,46]. Similar chronic inflammatory and remodeling effects have also been reported in terrestrial ecosystems, suggesting cross-system relevance.

### 3.4. Genotoxicity and DNA Damage Mechanisms

DNA damage represents one of the most concerning long-term consequences of MPs/NPs exposure due to its potential for causing mutations that could contribute to cancer development or heritable genetic changes. Although direct NP-specific genotoxicity evidence is limited in our dataset, recent mechanistic studies indicate NPs can exacerbate oxidative and organelle stress [26,27,28,33,36], plausibly amplifying DNA strand break burden. Oxidative stress-induced DNA damage appears to be the primary mechanism through which MPs/NPs cause genotoxicity, with multiple types of DNA lesions observed following particle exposure. The spectrum of DNA damage includes base modifications, strand breaks, and crosslinks, each with distinct mutagenic potential and repair requirements [47,48,49,50,51,52,53].

8-hydroxy-2’-deoxyguanosine (8-OHdG) is a widely used marker of oxidative DNA damage; elevated levels have been reported after MPs/NPs exposure and often correlate with other oxidative stress indicators Elevated 8-OHdG levels have been reported in cells and tissues exposed to various types of MPs/NPs particles. Liquid chromatography–mass spectrometry analyses have documented dose dependent increases in 8-OHdG formation following MPs/NPs exposure, with levels correlating with other markers of oxidative stress. The persistence of 8-OHdG lesions depends on the efficiency of base excision repair pathways, which may themselves be compromised by MPs/NPs-induced cellular stress [49,50]. Most direct evidence of DNA damage currently derives from studies on microplastics (e.g., oxidative lesions and strand breaks reported in [49,50,51]). Given that NPs can be efficiently internalized and elicit pronounced oxidative and organelle stress responses [26,27,28,33,36], NPs-related genotoxicity is biologically plausible but remains under-documented in our reference set; we therefore avoid over-attribution and explicitly classify [49,50,51] as MPs evidence, while flagging an NPs evidence gap.

DNA strand breaks (DSBs) are repaired through homologous recombination (HR) or non-homologous end joining (NHEJ). MPs/NPs-induced oxidative stress and inflammation may bias cells toward error-prone NHEJ, raising risk of chromosomal aberrations [33,36,49,50,51,52,53]. The γ-H2AX assay, which detects phosphorylated H2AX at sites of DNA double-strand breaks, is commonly used to assess MPs/NPs-induced DNA damage. Immunofluorescence microscopy reveals rapid formation of γ-H2AX foci following MPs exposure, with the number and persistence of foci correlating with particle concentration and exposure duration [51]. These lesions are typically repaired through HR or NHEJ; however, oxidative stress and inflammatory signaling triggered by MPs/NPs can impair HR fidelity and shift repair toward error-prone NHEJ, thereby enhancing genomic instability. Such repair pathway imbalance provides a mechanistic link between MPs/NPs exposure and long-term cancer risk.

The cellular response to MPs/NPs-induced DNA damage involves multiple repair pathways that may be compromised by the same oxidative stress and inflammatory conditions that cause the initial damage. Base excision repair (BER) represents the primary pathway for repairing oxidative DNA lesions such as 8-OHdG, but this system can be overwhelmed by high levels of damage or impaired by oxidative stress. Oxidative damage induced by MPs/NPs increases the burden on BER pathways. Evidence on whether key BER enzymes such as OGG1 and APE1 are consistently down-regulated is mixed; further primary studies are needed to clarify enzyme-level changes [52,53]. Direct NPs evidence remains scarce; however, their higher uptake efficiency and organelle-level stress responses make NPs-induced DNA damage a critical research priority.

### 3.5. Endoplasmic Reticulum (ER) Stress and Protein Homeostasis

ER stress represents an important but understudied mechanism of MPs/NPs toxicity that can have profound effects on cellular protein homeostasis and survival. MPs/NPs can disrupt ER function through multiple pathways, including direct physical interaction with ER membranes, calcium homeostasis disruption, and oxidative stress-induced protein misfolding. The accumulation of misfolded proteins in the ER lumen triggers the unfolded protein response (UPR), a complex signaling network that attempts to restore proteostasis but can lead to apoptosis if stress is severe or prolonged [54,55,56,57,58].

The UPR represents the primary cellular mechanism for responding to ER stress, involving three major signaling pathways: PERK (protein kinase R-like ER kinase), IRE1 (inositol-requiring enzyme 1), and ATF6 (activating transcription factor 6). Plastic particle (MPs/NPs) exposure can activate all three pathways, leading to reduced protein synthesis, enhanced protein folding capacity, and increased ER associated degradation. Western blot analyses have demonstrated time-dependent phosphorylation of PERK and eukaryotic initiation factor 2α(eIF2α) following PS-MPs exposure, with corresponding decreases in global protein synthesis measured by metabolic labeling. The splicing of XBP1 mRNA, a hallmark of IRE1 activation, has been observed within hours of PS-MPs exposure and persists for extended periods [56,57,58]. Recent studies demonstrate PS-NPs can trigger ROS-dependent UPR, leading to apoptosis and ferroptosis with organ-specific toxicity [56,57,58].

Chronic UPR activation can become maladaptive, leading to cellular dysfunction and apoptosis if ER homeostasis cannot be restored. The transition from adaptive to maladaptive UPRs may depend on particle characteristics, exposure duration, and cellular capacity to manage protein folding stress. CHOP expression, a proapoptotic transcription factor induced by chronic ER stress, increases significantly in cells exposed to PS-MPs (in aquatic models) for extended periods. This suggests that prolonged exposure may overwhelm cellular adaptive mechanisms, and trigger can culminate in apoptosis and, in some models, ferroptosis [57].

ER calcium stores play critical roles in protein folding, cellular signaling, and apoptosis regulation, making calcium homeostasis disruption a potentially important mechanism of MPs/NPs toxicity. PS-NPs exposure has been shown to induce calcium release from ER stores through both IP3 receptor activation and direct membrane perturbation. The depletion of ER calcium can compromise protein folding capacity and activate store-operated calcium entry pathways, leading to sustained elevations in cytoplasmic calcium that can trigger mitochondrial dysfunction and apoptosis [58].

### 3.6. Cellular Senescence and Aging Pathways

Cellular senescence is a plausible downstream outcome of sustained oxidative and inflammatory stress from MPs/NPs exposure, arising from sustained DNA damage, oxidative stress, mitochondrial dysfunction, and chronic inflammation. Senescence can be induced through multiple pathways, including DNA damage, oxidative stress, mitochondrial dysfunction, and chronic inflammation, all of which are associated with MPs/NPs exposure. The accumulation of senescent cells in tissues can compromise organ function and create pro-inflammatory microenvironments that promote age-related pathologies [59,60,61,62,63].

The p53/p21 pathway represents a major mechanism of senescence induction following DNA damage, with persistent DNA damage signals leading to permanent cell cycle arrest. MPs/NPs-induced DNA damage may activate this pathway, leading to accumulation of senescent cells in exposed tissues. In vivo models report increased p53 and p21 in proliferative compartments after chronic plastic particle exposure [61].

Senescent cells develop a senescence-associated secretory phenotype (SASP) characterized by secretion of pro-inflammatory cytokines, chemokines, growth factors, and matrix-degrading enzymes that can have profound effects on surrounding tissues [62,63]. The SASP can promote inflammation, tissue remodeling, and create a pro-carcinogenic microenvironment that may contribute to age-related diseases and cancer development. Proteomic profiling of conditioned media from particle-exposed senescent cells shows elevated SASP factors (e.g., IL-6, IL-8, MMP-3) [63]. Emerging reports suggest NPs may trigger premature senescence at lower concentrations than MPs, though systematic evidence is still limited. Table 2 provides a classification of MPs/NPs-induced health impacts.

**Table 2:** Proposed classification of MPs/NPs-induced health impacts.

Impact Category	Affected Systems	Representative Clinical/Biological Manifestations	Useful Biomarkers/Indicators	Representative References
Acute local effects	Intestinal epithelial barrier	Irritation, barrier disruption, transient inflammation	TEER change; local ROS; cytokines (IL-1β/IL-6)	[30,31,32,33,34,35,36,41,42,43]
Chronic oral effects	Renal/hepatic/intestinal tissues	Chronic inflammation, remodeling/fibrosis, dysbiosis	SASP factors; MMPs; microbiome shifts	[29,43,44,45,46,59,60,61,62,63]
Systemic distribution	Blood/lymph; organ deposition	Translocation/bioaccumulation; low-grade inflammation	Blood/tissue MPs; systemic cytokines: IL-1β, IL-6, TNF-α, IL-18	[9,28,30,31,32,36,41,64,65,66,67,68,69,70,71,72,73]
Immunological effects	Innate/adaptive immunity	Immune dysregulation; chronic activation	IL-1β; IL-18; TNF-α; NF-κB	[39,40,41,42,43,44]

## 4. Global Policy Responses and Regulatory Frameworks

### 4.1. European Union Leadership in Comprehensive Regulation

The European Union (EU) has established itself as the global leader in comprehensive MPs regulation through a series of scientifically rigorous and legally binding directives that address multiple aspects of plastic pollution [74]. The Single Use Plastics Directive (EU 2019/904) represents a landmark achievement in environmental legislation, targeting the ten most commonly found single-use plastic items in European waters, which collectively account for approximately 70% of all marine litter items [75]. This directive employs a differentiated approach based on available alternatives to extended producer responsibility (EPR) schemes that make producers finance litter clean-up, collection, transport, and awareness measures [75].

Commission Regulation (EU) 2023/2055 represents the world’s first comprehensive regulatory framework specifically addressing synthetic polymer microparticles intentionally added to products. The regulation restricts placing on the market microplastics “as a substance on their own or in a mixture in a concentration ≥ 0.01% by weight”, and sets definitions, derogations (e.g., industrial-site uses with containment), and criteria for degradability/solubility; its scope covers a wide range of products/mixtures including cosmetics, detergents, and fertilizers (articles are generally out of scope unless specifically covered by the restriction) [76]. Implementation is phased: some measures apply immediately (e.g., loose plastic glitter and rinse-off cosmetic microbeads), while others have transition periods up to ~12 years depending on use [76]. Scientific recommendations have also informed this regulation, highlighting potential health risks of MPs/NPs [77].

The economic implications of EU microplastic regulations have been carefully assessed through comprehensive impact assessments. Estimates indicate the restriction could avoid ~500,000 tons of microplastics over 20 years [76]; the SUP Directive’s EPR provisions shift costs of waste management and litter control to producers, creating incentives for sustainable product design [75,78] (the EPR cost scope and execution are specified by the Directive and subsequent guidance).

### 4.2. Taiwan’s Integrated Approach to Plastic Pollution

Taiwan’s approach to plastic pollution represents one of the world’s most innovative and comprehensive policy frameworks, demonstrating how sustained civil society engagement can drive transformative environmental change. The Taiwan Marine Waste (Debris) Management Platform, established in 2017 through unprecedented collaboration between eight environmental non-Governmental Organizations (NGOs) and Taiwan’s Environmental Protection Administration, created a novel governance model that integrates scientific research, policy development, and public engagement. This platform has facilitated evidence-based policy development through systematic monitoring of marine debris, stakeholder consultation processes, and pilot implementation of innovative solutions [79,80]. The platform started with eight NGOs and later expanded [79,80].

Taiwan’s plastic reduction timeline represents one of the world’s most ambitious national commitments to eliminating single-use plastics, with clear milestones and accountability mechanisms that ensure steady progress toward the 2030 goal. Plastic bag controls began in 2002; official sources report about 58% reduction in plastic bag use (from 3.435 billion to 1.43 billion bags) [81]. This outcome is documented in official EPA/MoENV releases [81] and reviewed in academic literature [82]. Subsequent policies have expanded to cover microbeads in cosmetics, single-use tableware, and beverage containers, with each phase building on previous successes [82].

The planned prohibition on manufacturing, importing, and selling key single-use plastics items by 2030 represents an unprecedented national commitment. Taiwan’s strategy recognizes that effective control requires a lifecycle approach from production to disposal, coupled with investment in alternative materials/technologies and green public procurement to help create stable markets [83].

### 4.3. North American Regulatory Approaches

The United States has adopted a more fragmented but increasingly comprehensive approach to microplastic regulation, with federal legislation focused on specific product categories while state governments lead in broader plastic reduction measures. The Microbead Free Waters Act of 2015 represents the most significant federal achievement to date, establishing a complete prohibition on manufacturing and distribution of rinse-off cosmetics containing plastic microbeads by phasing out manufacturing from 1 July 2017, and distribution from 1 January 2018 (one year later for OTC drug–cosmetics) [84,85]. The law’s success demonstrates the potential for targeted federal action when scientific evidence is clear, and industry alternatives are available.

State level initiatives have filled many regulatory gaps left by limited federal action, with California leading in comprehensive plastic reduction measures. Policy discussions have considered source–capture approaches for microfiber emissions during laundering; available analyses indicate that washer-installed filters can substantially reduce releases [86]. This technology forcing regulation could drive innovation in appliance design and create models for adoption in other jurisdictions. Additional state measures include extended producer responsibility programs, single-use plastic restrictions, and mandatory recycled content requirements that collectively create a patchwork of regulations that may eventually drive federal harmonization.

Canada’s approach to plastic pollution demonstrates sophisticated integration of scientific assessment, regulatory development, and international cooperation within a federal system. The designation of plastic manufactured items as toxic substances under the Canadian Environmental Protection Act provides broad regulatory authority for comprehensive controls. The Federal Plastics Registry represents an innovative approach to plastic lifecycle tracking, requiring producers to report quantities and types of plastic placed on the Canadian market annually [87,88]. This data collection mechanism enables evidence-based policy development and helps identify priority areas for intervention. Canada’s commitment to zero plastic waste by 2030 aligns with international best practices but faces implementation challenges due to the country’s federal structure requiring coordination across multiple jurisdictions.

### 4.4. G7 Countries’ Policies and Commitments

The Group of Seven (G7) nations represent some of the world’s largest economies and plastic producers, making their policy approaches particularly influential in addressing global microplastic pollution. While these countries have collectively committed to ending plastic pollution by 2040 through various declarations, their individual regulatory approaches demonstrate significant variation in scope, ambition, and implementation mechanisms [89]. Table 3 presents an analysis of five major policy approaches to microplastic pollution control.

Japan’s approach to microplastic regulation exemplifies the challenges of balancing industrial interests with environmental protection. In 2018, the Japanese Parliament unanimously approved legislation specifically targeting microplastic pollution, marking a dedicated national measure on this issue. However, this legislation contains no enforcement mechanisms or penalties for non-compliance, relying instead on voluntary industry cooperation. This voluntary approach reflects Japan’s broader regulatory philosophy but has raised questions about effectiveness. Despite these limitations, Japanese industry has demonstrated progress, with major cosmetic manufacturers voluntarily phasing out microbeads ahead of any mandatory requirements. The country’s commitment to the Osaka Blue Ocean Vision, which aims to reduce additional marine plastic pollution to zero by 2050, represents an ambitious long-term goal, though concrete implementation pathways remain unclear [90,91]. Japan’s recent launch of the Atlas of Ocean Microplastics (AOMI) in May 2024 demonstrates the country’s strength in scientific monitoring and data sharing, providing a global platform for harmonized microplastic monitoring that could inform evidence-based policy development [92].

South Korea represents a more proactive regulatory approach among Asian G7 partners. The proposed Special Law on the Reduction and Management of Microplastics, announced for deliberation in September 2024, would create a comprehensive national framework (proposal stage). This legislation goes beyond simple product bans to create an integrated management system including a national Microplastics Countermeasures Committee, mandatory safety standards for products containing primary microplastics, and emission limits for secondary microplastics from plastic and electronic products. The law’s definition of microplastics as solid plastic particles less than 5 mm in diameter that are insoluble in water aligns with international scientific consensus while distinguishing between primary and secondary sources. Products subject to regulation would include health functional foods, daily chemical products, quasi-drugs, and electrical/electronic equipment, demonstrating the law’s comprehensive scope. Public awareness research in South Korea has shown strong support for microplastic regulation, with willingness-to-pay studies indicating Seoul metropolitan residents would accept significant costs for microplastic removal from marine environments [93].

China’s Regulatory evolution reflects its dual position as the world’s largest plastic producer and a country facing severe environmental challenges. The 14th Five-Year Plan Action Plan for Plastic Pollution Control (2021–2025) represents a significant shift from previous approaches, establishing concrete targets for plastic waste management and pollution prevention [94,95]. By 2025, China aims to effectively curb plastic waste leakage into the natural environment, with specific measures including reductions in single-use plastic tableware (e.g., city-level 30% targets) and phased improvements in recyclability (e.g., toward 85% for plastic packaging by 2030). China’s ban on microbeads in cosmetics, with production prohibited from 2020 and sales banned from 2022, demonstrates the country’s capacity for decisive action when political will exists. The approach emphasizes circular economy principles, with mandatory waste classification systems implemented in 46 key cities and ambitious targets for domestic waste incineration capacity reaching 800,000 tons/day by 2025. However, enforcement challenges remain significant given China’s vast territory and complex governance structure, with studies indicating substantial gaps between policy ambition and implementation reality [95].

Russia’s announced in 2021 plans to fully ban single-use plastic products by 2024—an ambitious goal that has faced significant implementation challenges. The proposed ban would cover 28 categories of disposable plastic products, including straws, plates, cups, coffee capsules, and various packaging materials. Natural Resources and Environment Minister Alexander Kozlov emphasized a gradual implementation approach to allow production reorganization, recognizing the economic implications for domestic industries. However, Russia’s limited participation in international plastic pollution frameworks, including reluctance to engage fully with UN treaty negotiations, has raised concerns about the country’s commitment to comprehensive action. Environmental groups, particularly Greenpeace Russia, have urged the government to adopt more ambitious approaches aligned with global standards, specifically advocating for Russia to support comprehensive lifecycle approaches rather than focusing solely on marine pollution [96].

Italy’s implementation of EU plastic pollution directives has generated controversy due to national modifications that potentially weaken environmental protections. Legislative Decree No. 196/2021 transposed the EU Single-Use Plastics Directive but added exemptions for biodegradable and compostable plastics meeting specific standards (UNI EN 13432 [97] or UNI EN 14995 [98]), provided they contain at least 40% renewable raw material content, increasing to 60% by 2024. This deviation from EU requirements led to formal complaints from environmental organizations and scrutiny from European authorities. Italy’s approach reflects tensions between environmental goals and support for domestic bioplastics industries [99]. The country has implemented plastic packaging tax (postponed to 1 July 2026; EUR 0.45/kg) and actively promotes textile waste recycling to address microfiber pollution, recognizing the Mediterranean region’s particular vulnerability to plastic pollution. Italian research initiatives, including the MICROPLASMA project for integrated monitoring systems, demonstrate the country’s scientific contributions to understanding microplastic pollution dynamics [100,101].

Spain’s Law 7/2022 on Waste and Contaminated Soil for a Circular Economy establishes some of the most ambitious plastic reduction targets among G7 nations. The legislation mandates a 50% reduction in single-use plastics by 2026 and 70% by 2030 compared to 2022 baseline levels, with specific collection targets for plastic bottles increasing from 77% in 2025 to 90% by 2029. Unique provisions include requirements for retail stores over 400 m^2^ to dedicate at least 20% of floor space to packaging-free products and. restrictions on certain chemicals (e.g., phthalates; BPA) in food packaging. The 2024 plastic pellet spill along the Galician coast, which released millions of particles into marine environments, served as a catalyst for stronger enforcement and highlighted the need for comprehensive pellet loss prevention regulations. Spain’s approach demonstrates how environmental disasters can accelerate policy development and implementation [102].

The collective G7 commitments, including the 2018 Ocean Plastics Charter and the 2023 agreement to end plastic pollution by 2040, provide overarching frameworks for national action. However, significant variation across national approaches—from Japan’s voluntary compliance model to Spain’s mandatory targets—illustrates the challenge of harmonization. G7’s support for UN Global Plastics Treaty negotiations reflects the recognition that national actions alone cannot address this transboundary challenge. Success in achieving the 2040 goal will require not only strengthened national policies but also mechanisms for technology transfer, financial support for developing nations, and harmonized standards that prevent regulatory arbitrage while still respecting national circumstances Note: the 2018 Ocean Plastics Charter was launched under Canada’s presidency and was not signed by all G7 members [89,103,104].

## 5. Technological Solutions and Future Directions

### 5.1. Advanced Detection and Removal Technologies

Current analytical methods for MPs detection and characterization face significant limitations in sensitivity, specificity, and throughput that constrain both research and monitoring efforts. Development of rapid, cost-effective, and standardized analytical methods represents a critical priority for advancing the field [64,105]. Table 4 compares current and emerging technologies for MPs detection and removal.

Traditional methods such as visual identification and basic FTIR spectroscopy are being supplemented by advanced techniques including Raman microspectroscopy [105,106], pyrolysis gas chromatography–mass spectrometry [107], and automated FTIR-imaging workflows [64]. These technologies offer improved detection limits and chemical specificity but require substantial investments in equipment and expertise. Automated particle identification systems combining advanced imaging with artificial intelligence could dramatically improve throughput and consistency of MPs analysis, with recent machine-learning pipeline reporting high classification accuracy on benchmark datasets [108]. Stimulated Raman scattering (SRS) enables label-free single-particle imaging of NPs and complements Raman pipelines for submicron detection [9,106]. FTIR resolution is limited for NPs; complementary Raman, pyrolysis-GC–MS, or machine learning-based pipeline are needed [64,105,106,107,108].

Magnetic separation technologies have demonstrated remarkable potential for cost-effective MPs removal from water systems. Ferrofluid-based systems have been demonstrated to effectively extract polyester and other plastic particles from environmental samples by magnetic attraction, while leaving non-plastic materials largely unaffected [109]. This proof-of-concept highlights a promising direction for scalable MPs removal, though further work is needed to optimize efficiency and evaluate large-scale implementation.

Emerging materials such as mesoporous MOFs (e.g., UiO-66-NH_2_/P123) can achieve high removal under laboratory conditions for PS-NPs in water [110]. Electrocoagulation systems also show promise, removing high fractions of NPs under near-neutral conditions [111]. Biodegradable starch–gelatin sponges offer a renewable alternative, capturing up to 90% of NPs/MPs in real water samples, with some variation based on matrix complexity [112]. Broader reviews emphasize adsorption-based technologies—including sponges, aerogels, biochar, and activated carbon—as versatile options for removing both MPs and NPs from aquatic environments [113]. For NPs specifically, metal–organic frameworks (UiO-66-NH_2_/P123), electrocoagulation, and biopolymer sponges have shown promising removal efficiency [110,111,112,113].

### 5.2. Biological and Enzymatic Degradation

Biological approaches to MPs degradation represent promising long-term solutions that harness natural metabolic processes for polymer breakdown under environmentally relevant conditions. Genetically engineered bacteria and fungi demonstrate remarkable capacity for degrading various plastic polymers through enzymatic processes that could provide sustainable alternatives to physical and chemical treatment methods [114,115]. Recent discoveries include bacterial strains capable of utilizing PET as sole carbon sources, with growth rates approaching those on conventional substrates. The identification and characterization of plastic-degrading enzymes from environmental isolates have accelerated dramatically, with metagenomic approaches revealing previously unknown enzymatic diversity.

Enhanced PET-degrading enzymes can now function at temperatures approaching 70 °C and achieve high depolymerization yields at scale (e.g., ≥90% in ~10 h at ~72 °C) [116]. Engineered LCC variants show markedly improved activity suitable for industrial recycling [116]. In parallel, computational redesign using the GRAPE strategy produced the DuraPETase variant, which exhibits a 31 °C increase in melting temperature and >300-fold higher activity against semicrystalline PET under ambient conditions, representing a complementary advance in enzyme stability and efficiency [117]. Multi-enzyme systems such as PETase–MHETase demonstrate synergistic depolymerization of PET to its monomers (TPA, EG) [118]. In addition, machine learning-aided engineering has enabled the creation of FAST-PETase, which efficiently depolymerizes PET under mild temperatures (~30–50 °C) and supports closed-loop recycling [119]. Consolidated bioprocessing strategies are emerging, in which engineered microbes, such as *Ideonella sakaiensis*, can assimilate PET as a carbon source, forming the basis for subsequent metabolic engineering efforts for value-added bioconversions [120]. Lifecycle analyses suggest such biological recycling approaches could substantially reduce greenhouse-gas emissions relative to conventional routes when integrated with renewable energy and carbon capture [121].

### 5.3. Prevention and Source Reduction Technologies

The prevention of MPs requires a hierarchical approach progressing from passive end-of-pipe filtration to proactive material design. Using textile microfibers as a model system, this progression illustrates how technological interventions can evolve from reactive containment to fundamental prevention through innovative design.

Washing machine filtration systems represent one of the most promising approaches for preventing microfiber MPs release at the source during textile laundering, with the potential to reduce emissions through device- or membrane-based interception reported at up to ~87% in field/bench studies, and with certain surface-treatment strategies on specific materials achieving reductions up to ~95% under standardized test conditions when properly designed and maintained [122,123,124,125,126]. These systems intercept synthetic microfibers before they enter wastewater streams, preventing environmental contamination while enabling fiber recovery and potential recycling. Recent innovations include automated or cyclone-integrated filters that maintain high capture efficiency with reduced clogging and longer maintenance intervals [125], addressing a key barrier to consumer adoption. The integration of microfiber filters into washing machine designs from the manufacturing stage, rather than as aftermarket additions, could dramatically improve adoption rates and effectiveness [126].

Textile innovation and fiber engineering offer long-term solutions for reducing microfiber shedding through material properties rather than end-of-pipe treatment. Potential NPs generation during wear/wash remains under-measured. Advanced fiber surface treatments can reduce fiber breakage during mechanical stress, while alternative yarn construction techniques minimize loose fiber ends that contribute to shedding [127]. Recent developments include bio-based or chitosan-based coatings that strengthen fiber-to-fiber bonds without compromising textile properties, achieving high-percentage reductions (in some cases approaching ~95% for specific fabrics and test protocols) in microfiber MPs release during standardized testing [128,129,130]. Emerging polymer designs show reduced shedding in controlled laundry tests [130], while marine-degradable alternatives are also under development [131]. Although most data concern MPs, abrasion and laundering also plausibly generate NPs, which remain under-measured; design optimization should therefore address both MPs and NPs” [113,122,123,124,125,126,127,128,129,130].

Biodegradable synthetic fibers represent an emerging technology that maintains the performance characteristics of conventional synthetic textiles while showing biodegradation under marine conditions (e.g., marine-degradable poly(ester amide)s) [131,132,133]. Lifecycle assessment (LCA) explicitly incorporating microfiber emissions indicate that these materials could substantially reduce the environmental footprint of textile production while addressing MPs pollution at its source [65,66].

## 6. Clinical and Public Health Implications

### 6.1. Healthcare Professional Education and Training

The integration of environmental health topics including MPs and NPs exposure into healthcare professional education represents a critical need for protecting public health. Current medical, dental, and nursing education programs often provide inconsistent coverage of environmental determinants of health [67,68,69,70]. Given evolving evidence—including emerging human biomonitoring reports of polymer particles in blood and placenta—education should prepare clinicians for prudent risk communication and exposure-reduction counseling [5,71,72,134].

The development of competency-based educational modules that can be integrated into existing curricula represents a practical approach to addressing this gap. These modules should cover fundamental concepts of MPs and NPs sources and exposure pathways, cellular and molecular mechanisms of toxicity, measurement limits (particularly for NPs), clinical manifestations of exposure, and evidence-based prevention strategies [5,70,71,72,73,134,135]. Case-based learning approaches that present realistic clinical scenarios can help students develop skills in patient assessment and counseling. Interprofessional education opportunities that bring together students from medicine, dentistry, nursing, and public health can foster collaborative approaches to addressing this complex environmental health challenge.

Continuing education programs for practicing healthcare professionals could provide immediate capacity building for addressing current patients’ environmental health concerns. Online learning platforms offer scalable solutions for reaching large numbers of practitioners, with interactive modules, self-assessment tools, and clinical decision support resources. Authoritative reviews and guidance highlight substantial uncertainty—especially for NPs—so clinical resources should emphasize critical appraisal of methods and cautious interpretation rather than prescriptive screening recommendations [72,73,134,135]. At present, routine incorporation of MPs and NPs exposure assessment into general health screenings is premature; priorities include exposure source reduction and development/validation of biomarkers [5,71,72,73,134,135]. Curricula should emphasize limitations in NPs detection and avoid over-extrapolating MPs findings to NPs, while providing evidence-based language for patient communication [5,9,72,73,106,134,135].

### 6.2. Public Health Surveillance and Intervention

Although this review focuses on oral health implications, these findings must also be interpreted in the broader context of systemic exposure and public health. Population-level surveillance systems for MPs and NPs exposure and health effects could provide early warning of emerging health risks and data for policy development. Surveillance should be size-resolved (including NPs) and methods-transparent to avoid over-interpreting MPs-only datasets. Such systems should integrate environmental monitoring, size-resolved metrics, and health-relevant indicators, while recognizing that validated biomarkers for NPs remain limited relative to MPs [67,68,72,73,134,135]. Existing environmental health surveillance infrastructure could be adapted to include MPs and NPs monitoring, leveraging established sampling networks and analytical capabilities [5,71,72,73,134,135]. Biomarker development is a critical research need; early human biomonitoring reports (blood, placenta) illustrate feasibility but also current methodological constraints [72,73,134,135]. Community intervention programs that address MPs exposure at the population level could achieve greater impact than individual-focused approaches. These programs might include community-wide education campaigns, infrastructure and source reduction measures, and policy advocacy for local plastic reduction initiatives [67,68,69,70]. Participatory research approaches that engage community members in exposure assessment and intervention design can improve program relevance and effectiveness. Evaluation frameworks that assess both exposure reduction and health outcomes are essential for demonstrating program impact and informing scale-up decisions [67,68,69,70,72].

Health impact assessment methods adapted to MPs/NPs issues could support policy decision-making by quantifying potential health benefits of different intervention options. Co-benefits—such as reduced co-exposure to packaging-associated chemicals—should be accounted for, and quantitative risk models/economic analyses must transparently reflect current uncertainties (particularly for NPs) and be updated as new toxicology and exposure data emerge [69,72,73,135]. Surveillance indicators must be size-resolved (including NPs) and method-transparent; biomarkers for NPs remain scarce and require validation [9,67,68,72,73,134,135].

## 7. Conclusions

The evidence presented in this review demonstrates that MPs and NPs represent a significant potential risk to human health that demands immediate, coordinated action from all stakeholders in healthcare, research, policy, and industrial sectors. The oral cavity’s unique vulnerability as both a direct target of MPs assault and a gateway for systemic dissemination amplifies these health risks, creating a dual burden where local oral pathologies compound systemic toxicological effects. The convergence of ubiquitous environmental contamination, multiple exposure pathways, and well-documented cellular toxicity mechanisms creates an unprecedented public health challenge that transcends traditional disciplinary boundaries and requires innovative, integrated solutions.

The quantitative exposure assessment data revealing daily emissions of 0.36–74 billion particles from oral healthcare products alone represents a paradigm shift in our understanding of iatrogenic exposure risks in routine healthcare practices. The recent recognition of dental floss as a significant source of both MPs and PFAS exposure adds another dimension to this complex problem, with billions of floss picks entering waste streams annually while delivering chemical and particle contaminants directly to oral tissues. These findings challenge fundamental assumptions about the safety of products designed to promote health and highlight the need for comprehensive reassessment of materials used in healthcare. NPs exposures are plausible but currently under-quantified in oral-care products.

At the cellular level, our analysis reveals that MPs-induced and NPs-induced toxicity operates through multiple interconnected mechanisms including oxidative stress generation, inflammatory response activation, genotoxic damage, mitochondrial dysfunction, ER stress, and cellular senescence. These mechanisms do not operate in isolation but rather create cascading effects that amplify cellular damage and compromise tissue function. The oral cavity’s unique position as both a primary site of exposure and a gateway for systemic distribution makes understanding these mechanisms particularly crucial for protecting public health. The preclinical, preliminary evidence suggesting intergenerational effects via epigenetic mechanisms is emerging but limited and should be interpreted cautiously to address this challenge, as current exposures may affect future generations.

The analysis of global policy responses reveals encouraging progress toward comprehensive regulatory frameworks, with the EU demonstrating the potential for science-based, legally binding approaches to microplastic control. Taiwan’s comprehensive approach shows how sustained civil society engagement and political commitment can drive rapid transformation. The varying approaches among G7 nations, such as Japan’s voluntary compliance model and South Korea’s proposed comprehensive legislation, illustrate both the diversity of regulatory strategies and the need for greater harmonization. However, the fragmented nature of regulatory approaches in many jurisdictions creates implementation challenges and may limit overall effectiveness. The significant variation in enforcement mechanisms, ranging from legally binding EU regulations to voluntary Japanese guidelines, suggests that policy effectiveness depends not only on regulatory design but also on implementation capacity and political will.

The technological solutions reviewed demonstrate remarkable innovation potential, from magnetic separation systems achieving 87% removal efficiency to biodegradable capture materials with 99.9% effectiveness. Biological degradation approaches show particular promises for addressing the fundamental challenge of plastic persistence in environmental systems, while prevention-focused technologies offer opportunities to address MPs generation at source. The convergence of advanced materials, science, biotechnology, and engineering creates unprecedented opportunities for developing comprehensive solutions to MPs pollution.

The integration of MPs/NPs considerations into clinical practice and public health represents both a challenge and an opportunity. Healthcare professionals must be equipped with knowledge and tools to assess and address MPs/NPs exposure to their patients, while public health systems must develop surveillance and intervention capabilities appropriate to this emerging threat. The development of clinical guidelines, educational resources, and decision support tools will be essential for translating research findings into practice improvements.

Future research priorities should include development of standardized analytical methods for MPs/NPs detection and quantification in biological samples, with emphasis on clinically relevant matrices such as saliva, blood, and tissue biopsies. Long-term epidemiological studies are urgently needed to establish dose–response relationships and identify health outcomes associated with chronic MPs/NPs exposure. The development of biomarkers for exposure assessment and early biological effects could enable targeted interventions and monitoring of intervention effectiveness. Innovation in plastic-free alternatives for essential healthcare products represents both a research and commercial opportunity, with potential for significant health and environmental benefits. Assessment of intervention effectiveness at population scales will be crucial for informing policy decisions and resource allocation.

The urgency of the MPs/NPs threat requires that we act decisively on the basis of current knowledge while continuing to refine our understanding through ongoing research. The precautionary principle supports implementing protective measures now while additional studies continue to refine risk assessments and identify optimal intervention strategies. In particular, the relevance of NPs remains under-quantified due to analytical detection limits, underscoring the need for NP-focused exposure studies.

## Figures and Tables

**Table 3:** Comparative analysis of global MPs/NPs policy approaches.

Policy Approach	Scope	Implementation	Strengths	Limitations	Examples
Comprehensive frameworks	Entire plastic lifecycle	Phased, multi-year	Holistic approach Long-term vision Stakeholder engagement	Complex implementation High costs Requires coordination	EU, Taiwan
Product-specific bans	Single products/categories	Rapid deployment	Clear targets Easy enforcement Quick results	Limited scope May shift to alternatives Narrow impact	US (microbeads)
Extended producer responsibility	Production to disposal	Market-based	Economic incentives Industry innovation Self-sustaining	Enforcement challenges Cost pass-through Variable compliance	Germany, Republic of Korea
Voluntary measures	Industry self-regulation	Flexible timeline	Low resistance Industry cooperation Flexible approach	Limited effectiveness No enforcement Inconsistent adoption	Japan, Industry initiatives
Economic instruments	Taxes, fees, subsidies	Immediate	Revenue generation Behavior change Market signals	Regressive effects Administrative burden Avoidance potential	Spain (plastic tax), Taiwan (fees)

**Table 4:** Comparison of MPs/NPs detection and removal technologies.

Technology Type	Principle	Advantages	Limitations	Development Stage	Applications
Spectroscopic methods	FTIR, raman analysis	Chemical identification Non-destructive Established protocols	Time-intensive Size limitations Equipment cost	Commercial	Research, monitoring
Magnetic separation	Ferrofluid attraction	High efficiency Scalable Low energy	Polymer-specific Ferrofluid recovery Initial costs	Pilot scale	Water treatment
Biodegradable filters	Biomimetic capture	Sustainable High capture rate Biodegradable	Replacement frequency Production scale Flow rate limits	Development	Point-of-use filtration
Enzymatic degradation	Biological breakdown	Complete degradation Environmentally safe Self-sustaining	Slow process Polymer specificity Temperature sensitive	Research	Waste treatment
Machine filters	Physical retention	Immediate impact Consumer-friendly Proven technology	Maintenance required Partial capture Retrofit costs	Commercial	Washing machines

## Data Availability

Data will be made available on request.
